# Innovative interventions to promote positive dental health behaviors and prevent dental caries in preschool children: study protocol for a randomized controlled trial

**DOI:** 10.1186/1468-6708-14-118

**Published:** 2013-04-30

**Authors:** Xiaoli Gao, Edward Chin Man Lo, Colman McGrath, Samuel Mun Yin Ho

**Affiliations:** 1 Dental Public Health, Faculty of Dentistry, The University of Hong Kong, Prince Philip Dental Hospital, 34 Hospital Road, Sai Ying Pun, Hong Kong, SAR, China; 2 Department of Applied Social Studies, City University of Hong Kong, Tat Chee Avenue, Kowloon, Hong Kong, SAR, China

**Keywords:** Motivational interviewing, Caries risk assessment, Oral health intervention, Oral health education, Dental counseling, Children, Preschool, Parents, Randomized controlled trial

## Abstract

**Background:**

Dental caries (tooth decay) is highly prevalent and is largely attributable to unhealthy self-care behaviors (diet and oral hygiene). The conventional (health) education (CE), focusing on disseminating information and giving normative advice, often fails to achieve sustained behavioral changes. This study incorporates two innovative elements into CE: (i) motivational interviewing (MI), a client-centered counseling for changing behaviors, and (ii) an interactive caries risk assessment (RA) tool, which is devised to facilitate dental counseling and may enhance MI in several ways. Through a randomized, controlled, evaluator-blinded trial, three intervention schemes (CE, CE + MI, and CE + MI + RA) will be compared for their effectiveness in eliciting dentally healthy behaviors and preventing caries in preschool children.

**Methods/Design:**

This study targets 3-year-old children who are at a critical stage for embedding health habits. Children with unfavorable dental behaviors (insufficient toothbrushing and/or frequent snacking) and their parents will be recruited from 12 participating kindergartens. Parent-child dyads (n = 690) will be randomly assigned into three groups. In the first group (CE), oral health information and advice will be delivered to parents through pamphlets. In the second group (CE + MI), in addition to the pamphlets, individual MI counseling with each parent will be performed by one of two trained dental hygienists. In the third group (CE + MI + RA), besides pamphlets and MI, interactive RA will be integrated into MI to motivate parents and facilitate their informed decision making and goal planning. At baseline and after 12 and 24 months, parents will complete a questionnaire and children will undergo a dental examination. The effectiveness of the intervention schemes will be compared over 12 and 24 months. The primary outcome will be caries increment in children and proportion of caries-free children. Secondary outcomes will be changes in parental efficacy for protecting children’s oral health and changes in children’s dental behaviors.

**Discussion:**

Motivating and empowering parents to cultivate dentally healthy habits of young children presents both promises and challenges. With careful methodological considerations, this study is expected to provide scientific evidence for public health workers, dentists, and dental auxiliaries (nurses and hygienists) to choose appropriate interventions to advance children’s oral health.

**Trial registration:**

HKCTR-1455

## Background

Dental caries (tooth decay) is one of the most common oral diseases. As estimated by the World Health Organization (WHO), 5 billion people of the world’s 6.5 billion population are affected by dental caries [1]. The onset of dental caries can start soon after an infant's teeth erupt. The presence of one or more decayed, missing (due to caries), or filled tooth surfaces in any primary tooth in a child 71 months of age or younger is defined as early childhood caries (ECC) [2]. In the USA, caries is the most common chronic childhood disease, five times more prevalent than asthma, afflicting 30% of children aged 2 to 5 years [3]. In Hong Kong, 50% of children suffer from caries at the age of 5 years [4]. ECC imposes significant threats to the physical, psychological and social well-being of young children, constitutes a heavy financial burden on society, and, if not treated promptly and properly, may cause lethal systemic infections [5,6]. Recent life-course studies have linked ECC to subsequent caries in permanent dentitions [7,8], indicating a lifetime impact of ECC on one’s health.

Like many other chronic diseases, dental caries is a multifactorial disease highly determined by one’s ‘lifestyles’ [9,10]. It is preventable by adopting healthy behaviors, such as regular toothbrushing, favorable dietary habits and regular dental check-up. Promoting dentally healthy lifestyles is identified by the WHO as a priority and strategic orientation for oral health promotion [11]. Since early childhood is a critical stage for forming health habits, and parents are often receptive at this stage [12], this period offers a unique opportunity for behavioral interventions. Cultivating dentally healthy habits among preschool children, whose permanent teeth will later erupt, would maximize the chance of a caries-free permanent dentition throughout a lifetime [2].

Conventionally, health education focuses on disseminating information and giving normative advice. The insufficiency of conventional (health) education (CE) has been documented [13]. Although dental knowledge can almost always be improved by CE, such knowledge gain does not translate into sustained changes in dental behaviors [13]. A typical CE session is often an exercise in overt persuasion. However, what appears to be a convincing line of reasoning to the dental professional falls on deaf ears or result in reluctance to change [14]. The fruitless efforts of CE have led initially enthusiastic dental professionals to a state of burnout and created skepticism toward such attempts [14,15].

To address the limitations of CE, motivational interviewing (MI) was developed as an interventional style. Evolving from Rogers’ person-centered counseling approach and embracing the transtheoretical theory, MI elicits clients’ intrinsic motivations, enhances their readiness to change, and helps to explore and resolve ambivalence [16]. Clients assess their own behaviors, present arguments for change and decide what behavior, if any, to focus on, while the counselor helps to create, by skilful questioning and reflection, an acceptable resolution that triggers change [17]. Such a client-centered approach is in clear contrast to the traditional health education and counseling in which professionals are the most active participants in presenting problems and offering solutions, while clients are normally excluded from problem definition and decision-making [17,18].

MI was first applied to tackle the most difficult-to-change behaviors - substance abuse - and was later used to treat a broad range of lifestyle problems, such as eating disorder, lack of physical exercise, and poor adherence to medication regimens. Its treatment effect ranged from 0.25 to 0.57 across studies [19]. A systematic review has shown that, in 60% of 29 randomized trials, MI yielded at least one significant behavioral change [20]. MI did not emerge in dental research until recent years. In a South Asian immigrant community in Canada, a dental MI intervention that focused on modifying mothers’ infant-rearing practices was found to be effective in reducing the caries increment in infants (6 to 18 months of age) over 2 years (hazard ratio = 0.54, 95% confidence interval = 0.35 to 0.84) [15,21]. Nevertheless, in a recent study among low-income African Americans, MI with caregivers showed no significant effect on children’s dietary habits and caries increment; only marginal improvement was seen in their oral hygiene measures which, however, declined over 2 years [22]. The limited number of dental MI studies and their contradictory findings point to the need for further investigations involving a breadth of populations for understanding the role of MI in improving children’s oral health.

**Figure 1:**
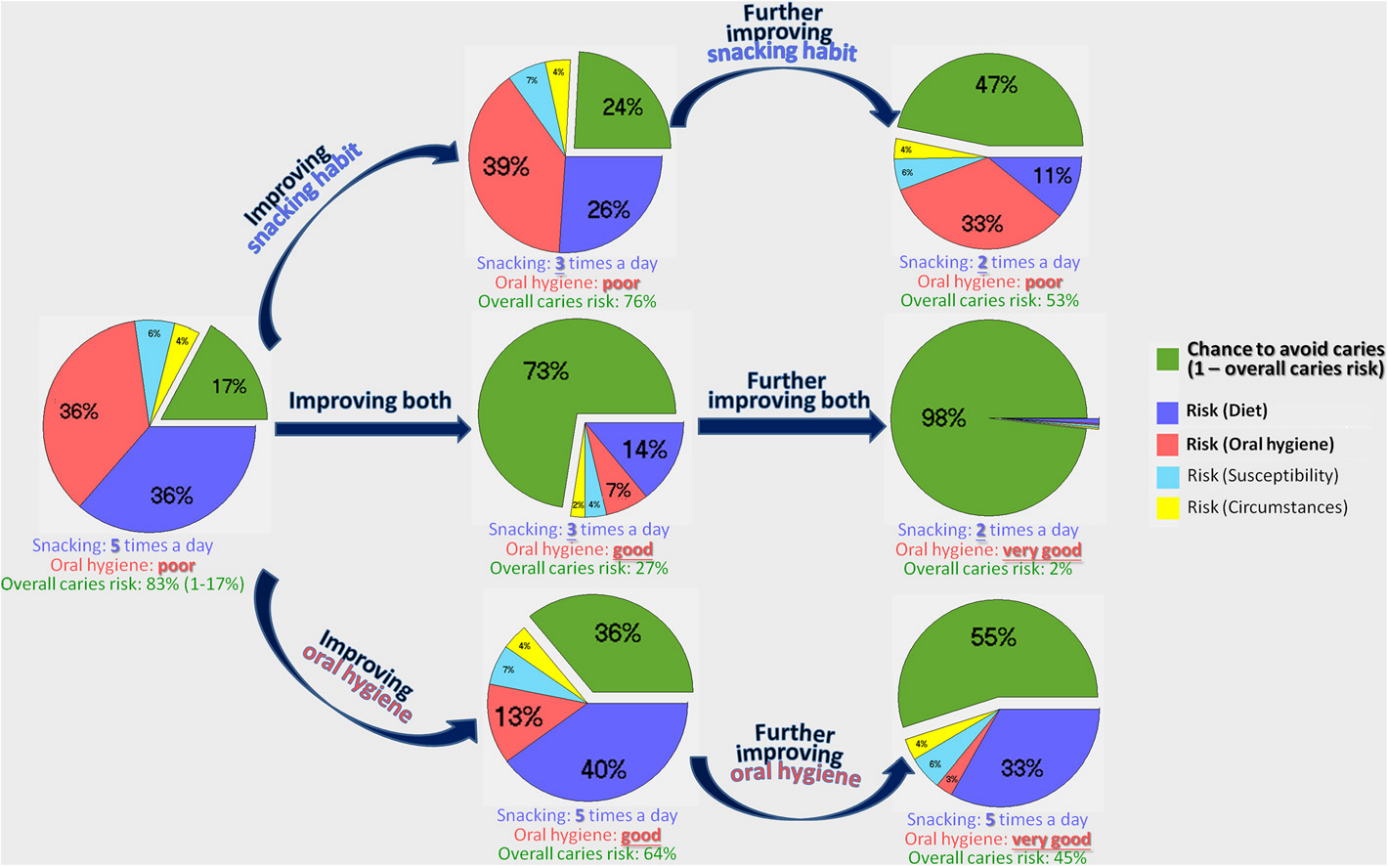
A case exemplifying the interactive risk assessment (Current risk and risk reduction through various behavioral changes are demonstrated).

To maximize the potential of MI, some tools can be incorporated to facilitate the MI counseling. In the dental context, a potentially useful tool to assist MI counseling is an interactive caries risk assessment (RA) program called Cariogram [23]. Enhanced with artificial intelligence, this computer program generates a pie diagram that analyses one’s caries risk in a manner understandable to lay persons. The overall risk is quantified and broken down into various causes, such as diet and oral hygiene. In contrast to a standard list of do’s and don’ts prescribed to all, Cariogram pinpoints the most needed behavioral change(s) to a specific client, while allowing alternative options. The dental counselor can discuss with the client about various possible behavioral changes, whose expected health gains (risk reduction) can be demonstrated interactively (Figure 1).

This interactive RA program may facilitate MI in several ways: (1) help to establish rapport and bring the client into a meaningful conversation; (2) assist the client’s systematic reflection on and appraisal of his/her dental habits; (3) motivate the client by demonstrating his/her caries risk and health gains achievable through behavioral changes; (4) pinpoint target behavior(s) and offer alternative solutions; (5) allow stepwise improvement and enhance self-efficacy; and (6) facilitate better informed decision-making and assist the client to set his/her own goal and agenda. Theoretically, this interactive RA program may enhance the effectiveness of MI in changing dental behaviors. This assumption, however, is yet to be empirically tested.

This study, through a randomized controlled trial, aimed to evaluate and compare the effectiveness of three intervention schemes (CE, CE + MI, and CE + MI + RA) in enhancing parental efficacy for protecting children’s oral health, improving preschool children’s dental behaviors, and preventing early childhood caries. The null hypothesis is: There is no difference among these three groups in parental efficacy, children’s dental behaviors, and caries increment among children.

## Methods/Design

### Sample size calculation

The main outcome of this study will be any caries increment or not (change in decayed, missing, and filled teeth (△dmft >0 or = 0) in the study children. Our latest survey showed that about 35% of Hong Kong children aged 3 years develop new caries in one or more teeth (△dmft >0) in a year [24]. An intervention will be considered to have a clinically significant effect if it reduces this rate to 20% in a year. Such a magnitude of effect size (relative risk of 0.57) was also reported by a previous MI trial in infants [15]. In order to detect such a reduction at a significance level of 5% and a statistical power of 80%, 184 parent-child dyads per group will be needed (multiple comparisons considered). Allowing for a 20% attrition rate, 230 parent-child dyads would need to be recruited into each of the three study groups. The total sample size will be 690.

### Recruitment and randomization

The protocol of this study has been reviewed by the Institutional Review Board of University of Hong Kong⁄Hospital Authority Hong Kong West Cluster. An ethical approval was obtained (#UW 11-483). This study involves parent-child dyads. Informed written consent will be obtained from each participating parent.

To be eligible to join this study, a child should be (a) enrolled in kindergarten Grade 1 (K1), (b) aged 3 years, and (c) having unfavorable oral health behavior(s) (that is, a child who needs intervention). Unfavorable oral health behaviors are defined as “brush teeth less often than twice a day” and/or “snack three times or more a day”. Each child will be required to join this study together with his/her parent (mother or father) who spends the most time with him/her, so that a parent-child dyad can be recruited. A child will be excluded if (i) he/she has a serious medical condition or (ii) his/her parents are non-Chinese speaking and cannot understand the oral health materials and counseling, which will be in Chinese.

A screening questionnaire will be used to identify participants who fulfil the above-mentioned criteria. According to our latest survey in Hong Kong [24], 60% of children aged 3 years have unfavorable oral health behaviors (24% with insufficient toothbrushing only; 26% with frequent snacking only; and 10% with both). To reach the target number of 690 parent-child dyads (230 dyads in each of the three groups), 1,150 children would need to be screened (1,150 × 60% = 690). Considering an average number of 120 children in K1 of a kindergarten and an estimated response rate of 80% (based on our recent experience), 12 participating kindergartens will need to be recruited (120 × 80% × 12 = 1,152).

A research nurse will review the screening questionnaire and recruit all eligible parent-child dyads who agree to participate. After baseline data collection, participants in each kindergarten will be randomly assigned into three groups (1, CE; 2, CE + MI; and 3, CE + MI + RA) by a research assistant not involved in the enrolment of the participants, delivery of intervention, or data collection. The randomization will be stratified by parental education (“secondary education or below” or “tertiary education”) and child’s caries experience at baseline (“dmft = 0” or “dmft >0”), which are perceived important confounders in this study. For each stratum, to ensure an equal number of subjects in each group (that is, an allocation ratio 1:1:1), a block randomization will be performed (block size of 6). A computerized random sequence generator will determine intervention status of the subjects. Intervention status is then placed into a sequentially numbered envelope for each group and each envelope is sealed and opened before the delivery of intervention so that allocation concealment can be achieved.

### Oral health assessment

All participants will be assessed at baseline and followed-up after 12 and 24 months, so that the outcomes of the interventions can be determined.

A questionnaire (in Chinese) pre-tested in our previous study [24] will be completed by parents to gather information on (i) family socio-demographic background, (ii) parental knowledge of and attitudes toward oral health, (iii) parental efficacy in protecting the child’s oral health, (iv) child’s oral health behaviors (diet and oral hygiene), and (v) child’s history of dental treatment and use of caries-preventive agents, such as topical fluorides.

All child participants will undergo a dental examination. The examiner has been trained regarding the diagnostic criteria and examination methods and will be further calibrated against an experienced oral epidemiologist to ensure an agreement rate over 90%. Duplicate examinations will be carried out on 10% of the subjects, for assessing the intra-examiner reliability. Each child will be examined in the supine position. A mouth mirror, illuminated by an intra-oral LED light, and a community periodontal index (CPI) probe will be used. The status of each tooth surface will be assessed by visual inspection, aided by tactile inspection if necessary. No radiographs will be taken. The WHO recommended diagnostic criteria for dental caries will be followed - that is, caries will be recorded as present when a lesion in a pit or fissure, or on a smooth tooth surface, has an unmistakable cavity, undermined enamel, or a detectably softened floor or wall [25].

The oral hygiene status will be evaluated using the Silness-Löe Plaque Index [26]. The examiner will be blinded to the group allocation of each child subject throughout the whole study.

### Training counselors

Two dental hygienists will be trained to deliver counseling to the second group (CE + MI) and third group (CE + MI + RA). Training materials have been prepared by referring to key MI literature and guide books [17,18], a dental MI workbook [27], and the interactive RA program (Cariogram) manual [23]. The training will be delivered by an expert panel composed of a health psychologist experienced in MI counseling and two public health workers specialized in dental interventions and caries risk assessment. The 12-hour training will be customized to involve key caries-related behaviors and typical scenarios commonly encountered in the dental settings. Training sessions will include lectures, discussions, demonstrations, and role plays. A hygienist will proceed to contact subjects only after demonstrating satisfactory competency as assessed by the panel using the Motivational Interviewing Treatment Integrity (MITI) Coding System [28] and a skill evaluation scheme on using Carigoram.

In each of the two groups (CE + MI and CE + MI + RA), participants will be randomly divided into two halves, who will be interviewed by the two counselors, respectively. To ensure the counseling is being delivered consistently with high fidelity to the MI principles, all counseling sessions will be audio-recorded and 20% of randomly selected sessions will be continuously reviewed and rated using the MITI coding system.

### Behavioral interventions

At baseline, different groups (CE, CE + MI, and CE + MI + RA) will receive their respective interventions.

(1) Conventional (oral health) education (CE)

The CE will be delivered to each parent through three pamphlets titled “Toothbrushing - your child can do it”, “Child’s diet and dental health”, and “Oral health care for your children” [29]. These color printed materials are in Chinese and were designed by the Oral Health Education Unit of the government Department of Health. Besides textual messages, there are pictorial illustrations for readers to better understand the information and advice.

(2) Motivational interviewing (MI)

The MI will be performed in an undisturbed room in the kindergarten. A typical MI counseling session will last for 15 to 30 minutes and will contain the following elements:

(i) Establish rapport: The MI session shall begin with showing concern and getting the parent to talk. The counselor may ask about the child’s kindergarten life, daily routines at home, family’s dental health, their contact with dentists and dental experience. The counselor may also encourage the parent to talk about his/her understanding about oral diseases and their impacts on one’s daily life.

(ii) Self-assess behaviors and identify discrepancy: The counselor will ask open-ended questions, listen carefully and encourage the parent to talk about the child’s dental habits and the parent’s dental wants/desires for the child, thereby identifying the discrepancy between the child’s present behaviors and important goals (in this case, the child’s oral health).

(iii) Elicit need for changes and promote self-efficacy: Once the counselor has identified the parent’s self-motivation for changing the child’s behavior(s), the counselor will explore and subtly encourage the changes that need to be made. The counselor will affirm the parent’s competence and encourage additional self-motivational statements (change talks). The counselor will also encourage the parent to talk about the difficulties in changing the child’s habits (the child’s temperament and resistance, parenting stress, time constraint, other family members’ influence, etc.) and explore possible solutions.

(iv) Present options and set goals: Once the parent shows a desire to improve the child’s behavior(s), the counselor will discuss the options, try to elicit commitment from the parent, and encourage him/her to talk about what change he/she would like and feels confident to try. Whenever the parent is ready for goal setting, a number of possible plans will be discussed in detail, so that a specific, measurable, and realistic goal can be set.

The whole MI session will be non-judgmental, respecting the parent’s expertise on his/her own child. The counselor shall avoid giving premature advice and be sensitive to the signals of resistance, such as arguing, interrupting, blaming others, and inattention. These signals imply that the parent is not ready to change at that moment and useful strategies will be to emphasize choices, avoid arguing, or recognize that the parent has a valid point.

(3) Interactive risk assessment (RA)

To obtain a reliable caries risk assessment result, children in the CE + MI + RA group will receive biological tests on saliva buffering capacity and abundance of caries-associated bacteria, *mutans Streptococci* and *Lactobacilli*[23]. Refraining from foods, drinks, and toothbrushing for at least 1 hour, children will be instructed to chew on a paraffin pellet for 5 minutes so that stimulated saliva can be collected. Dentobuff Strip, Dentocult SM Strip, and Dentocult LB kits (Orion Diagnostica, Finland) will be used and standard procedures will be followed in processing the samples. Before interviewing a parent in the CE + MI + RA group, the hygienist will gather the information previously collected through the questionnaire, oral examination, and biological tests, and enter it into the Cariogram program for caries risk assessment.

The RA is to be nested into the MI counseling and can be introduced at different stages of the MI session, depending on the parent’s response: (a) if the parent is not actively involved in the conversation, the interactive program can be used to break the ice, explain dental caries and its causes, and bring the parent into meaningful discussions; (b) after rapport is established, if the parent shows difficulty in recalling/assessing the child’s dental behaviors, the program can serve as a framework to assist his/her reflection and help him/her to identify the discrepancy between the child’s dental habits and parental expectation; (c) if the parent expresses willingness to make a change, the counselor can discuss with him/her the behavioral change(s) that would benefit the child the most and demonstrate how much risk reduction can be achieved; (d) if the parent feels the child is not ready for the suggested behavioral change, other options can be provided in a ‘menu’ format, listing all possible behavioral changes in various magnitudes and presenting their respective health gains. This approach would enable the child to make a start, which may have a knock-on effect leading to further improvements [30]; (e) the strategic use of the program enables the parent to make better informed decisions and set his/her own goal and agenda (what to change and to what extent).

As shown in Table 1, for both the CE + MI and the CE + MI + RA groups, after initial counseling, two brief follow-up telephone calls will be made to parents in these two groups to assist in the preparation for change, encourage the start, and discuss difficulties and possible solutions. To maintain the behavioral change and avoid relapse, each parent will be telephoned three more times up to 6 months after the initial contact [15].

**Table 1:** Intervention activities (for CE + MI and CE + MI + RA groups)

**Activity**	**Time**	**Goals**
Initial counseling (X1)	Start of study	Establish rapport, discuss need and options, use strategies that structure and elicit change, and set goals
Follow-up telephone calls (X2)	2 weeks and 1 month after initial counseling	Assist preparation, encourage start, and solve problems
Maintenance telephone calls (X3)	2, 4, and 6 months after initial counseling	Promote maintenance, avoid relapse, and help re-establish change, if needed

CE, conventional (health) education; MI, motivational interviewing; RA, (caries) risk assessment.

### Benefits and risks of participation

Each participating child will receive a stationery kit (pencil, eraser and ruler costing around US$2) upon completing each oral health examination. The same stationery kit will also be given to each child who joined the screening. No monetary incentive will be provided. A written report on each child’s oral health status will be issued to his/her parents after each oral examination. At the end of the study, the children’s decayed teeth will be restored free of charge.

The risk of participating in this study is minimal, similar to that of routine dental checkups. No invasive procedures will be involved. Disposable materials and sterilized instruments will be used. Adverse events, if any, will be recorded and reported.

### Statistical analysis

Participants in the three intervention groups will be compared on their demographic background (age and gender), family socioeconomic status (parental education and family income), and baseline oral health behaviors and tooth status. The effectiveness of the intervention schemes will be evaluated and compared over 12 and 24 months through an intention-to-treat analysis, in which participants will remain in their originally assigned group regardless of the intervention actually received and subsequent withdrawal or deviation from the original intervention protocol [31]. For those who are lost to follow-up, the last assessment made will be considered valid throughout the study period.

The primary outcome will be caries increment in children and the proportion of caries-free children, measured by the number of new carious teeth (△dmft; continuous variable) and whether there are any new carious teeth (△dmft >0 or = 0; dichotomous variable). Secondary outcomes will be changes in parental efficacy in protecting children’s oral health and changes in children’s dental behaviors (outcome variables described in Table 2). Effect sizes (relative risk) and their confidence intervals will be calculated. Chi-square tests will be used for comparing proportions; means will be compared through parametric or non-parametric methods, as appropriate. Number needed to treat, calculated as 100 divided by the risk difference expressed as a percentage [32], will be presented to provide an estimate of the number of parents needed to be counseled in order to avoid one child with new dental caries.

**Table 2:** Outcome variables

**Outcomes**	**Variables**	**Codes**	**Source of information**
**PRIMARY OUTCOME**			
Caries increment in child*			
	Number of new carious teeth (△dmft)	Continuous	Dental examination
	Any new carious teeth (△dmft >0 or = 0)	Dichotomous	Dental examination
**Caries free child**	dmft = 0 or >0	Dichotomous	Dental examination
**SECONDARY OUTCOMES**			
Change in parental efficacy**			
Diet	View on the statement “I can control my child from frequent snacking even when he/she cries for it.”	(1) Decreased;	Questionnaire
		(2) No change;	
		(3) Increased	
Oral hygiene	View on the statement “I can make sure my child’s teeth are brushed thoroughly twice a day even when I am very busy.”	(1) Decreased;	Questionnaire
		(2) No change;	
		(3) Increased	
**Change in child’s dental behavior**			
Diet	Frequency of snacking per day	(1) Decreased;	Questionnaire
		(2) No change;	
		(3) Increased	
Oral hygiene	Frequency of toothbrushing per day	(1) Decreased;	Questionnaire
		(2) No change;	
		(3) Increased	
	Silness-Löe Plaque Index	Continuous	Dental examination

* dmft refers to decayed, missing, and filled teeth.

**Parental efficacy was measured using five-point Likert scale questions (“strongly agree”, “agree”, “neutral”, “disagree”, and “strongly disagree”).

## Discussion

To the best of our knowledge, this trial is the first attempt to investigate the effectiveness of the innovative interventions (MI and RA) in advancing preschool children’s oral health. Unlike previous dental MI studies targeting infants whose oral health is predominantly taken care of by their parents [15,21,22], this study focuses on preschoolers who are at a stage of forming their own health habits [12]. While the impact of early intervention (from infancy or even prenatal) is supported by sizeable scientific evidence [15,21,33,34], preschool age represents another window for behavioral intervention, because children’s food preference is being shaped [35], their toothbrushing habits are being reinforced [4], and parents are often receptive to health messages during this period of time [9,36]. Interventions in this age group are both promising and challenging. While dentally healthy behaviors cultivated in preschoolers reduce the caries risk in their future permanent teeth [37], the success of intervention requires adequacy in both steps, namely motivating and empowering parents and, through them, instilling and reinforcing favorable behaviors in their young children.

Randomization will be performed at the individual level in this study (that is, each parent-child dyad as a unit). Although children in the same class or kindergarten may be assigned to different groups, the possibility of intervention contamination is considered low. In most families in this metropolitan city with a fast-pace lifestyle, both parents are working and children are often sent to and fetched from school by domestic helpers or other carers. Parents of different children rarely meet and are often not close to one another, reducing the chance of intervention contamination. Besides, since MI and interactive risk assessment are highly individualized, the possibility and impact of contamination should be low. On the other hand, randomization at the individual level is preferable to cluster randomization at the class or kindergarten level as far as the comparability of groups is concerned, since children in different kindergartens and classes may differ in their demographic and socioeconomic background, health habits and dental caries rate.

Since behavioral change is the target of the interventions tested in this study, cognitive/behavioral outcomes are included in this study, along with the clinical outcomes. Although the former are self-reported, subjective and vulnerable to the Hawthorne effect [38], the combination of both ensures a certain objectivity of our outcome measures and allows us to explore the mediating behaviors of the clinical effect, if any. A report on the child’s tooth status and treatment need will be given to his/her parents after each dental examination. This may raise an issue of possible co-intervention given by dentists they visit in the study period. However, our past experience of conducting a dental program in this population showed that very few parents brought their child to see a dentist after receiving such reports and the treatments received were almost exclusively curative (restoration or extraction) with nearly no preventive care. Therefore, the confounding impacts of these dental visits are likely to be very small.

Two dental hygienists will serve as MI counselors in this study, while in some other studies dental MI was delivered by professional counselors (psychologists or social workers) [39,40]. Delegating the dental counseling work to professional counselors, who require minimal training on health MI, will definitely contribute to an easy implementation of the project. However, training dental professionals to be counselors attaches much higher applicability of our interventions to the real world dental setting. Besides regular review on the audio-recorded MI sessions, a well established coding scale MITI will be used to monitor and assess the fidelity of our MI intervention. This approach, rarely adopted in dental MI research, is highly desirable to ensure the quality of MI intervention.

MI represents a total reconceptualization and a radical change in dental intervention. Skepticism exists among professionals regarding the practicality of MI in the dental setting. Our preliminary study has shown that dental personnel could become competent MI counselors through short training and practice, and MI can be delivered through brief counseling sessions [41]. This supports the practicality of dental MI and its adoption in clinical practice once its effectiveness is established.

This study is expected to provide scientific evidence for dental professionals to choose appropriate interventions. The findings will directly contribute to the prevention of early childhood caries. Since diet and personal hygiene habits are also essential for combating other chronic dental diseases (for example, periodontal disease) and systemic diseases (for example, obesity and diabetes) [42], the intervention scheme may also contribute to advancing general health.

## Trial status

Kindergartens are being contacted. Participant recruitment will start soon.

## Abbreviations

CE: Conventional (health) education; dmft: Decayed missing, and filled teeth; ECC: Early childhood caries; K1: Kindergarten Grade 1; MI: Motivational interviewing; MITI: Motivational Interviewing Treatment Integrity; RA: (caries) risk assessment; WHO: World Health Organization.

## Competing interests

The authors declare that they have no competing interests.

## Authors’ contributions

XG conceived of this study, coordinated the study design and drafted this manuscript. ECML contributed to the study design, sample size calculation, and preparation of the manuscript. CM participated in the study design and contributed to the manuscript; SMYH provided expert inputs to the design of the psychological intervention and contributed to the manuscript. All authors have read the manuscript and approved the version submitted for publication.
